# Contrast-enhanced ultrasound of collecting duct carcinoma in the renal: a case report misdiagnosed as renal abscess on computed tomography scan

**DOI:** 10.3389/fonc.2024.1511009

**Published:** 2025-01-09

**Authors:** Yunhao Luo, Jing Zhang, Minggang Wu, Qiuyun Huang, Fangqin Liu, Lang Qiao

**Affiliations:** ^1^ School of Medical and Life Sciences, Chengdu University of Traditional Chinese Medicine, Chengdu, China; ^2^ Department of Ultrasound, Sichuan Integrative Medicine Hospital, Chengdu, China; ^3^ Department of Ultrasound, The Second Affiliated Hospital of Chengdu Medical College, Nuclear Industry 416 Hospital, Chengdu, China

**Keywords:** collecting duct carcinoma, Bellini duct carcinoma, contrast-enhanced ultrasound, CEUS, Sonazoid

## Abstract

Collecting duct carcinoma (CDC), also known as Bellini duct carcinoma, is a rare malignancy with significant challenges in early diagnosis. This paper presents a case report of CDC that was misdiagnosed as renal abscess on computed tomography (CT). A 49-year-old male patient was admitted to the hospital with bilateral lumbar pain, exacerbated on the left side, accompanied by hematuria. Following laboratory tests, CT scan, ultrasound, and contrast-enhanced ultrasound (CEUS), the CT diagnosis was renal abscess, whereas CEUS suggested a malignant tumor. After a multidisciplinary discussion, the patient underwent surgery and was diagnosed with CDC. Previous studies indicate that two-dimensional ultrasound of CDC often lack typical features, while CT and magnetic resonance imaging (MRI) scan usually show mild enhancement. CEUS has a unique advantage in visualizing the microvasculature, which aids in the diagnosis of CDC.

## 1 Introduction

Collecting duct carcinoma (CDC), a rare epithelial-derived tumor that originates from the collecting ducts (Bellini ducts) of the renal medulla, accounts for only 0.4% to 2.0% of all renal carcinomas (1, 2). The average age of onset for CDC is 55 years, with a male-to-female incidence ratio of about 2:1 (3). CDC is a highly malignant disease with complex clinical manifestations, and at the time of presentation, 30%-50% of patients have already developed distant metastases (1, 4). Therefore, early diagnosis of CDC is of great importance. Currently, there is a lack of characteristic imaging diagnostic criteria for CDC. Sonazoid contrast-enhanced ultrasound (CEUS) is a novel technology for the diagnosis of renal tumors (5). Compared to the commonly used Sonovue contrast agent, Sonazoid has higher mechanical index stability, allowing for longer observation of tumors (6). Compared to CT/MRI, CEUS has the advantages of being real-time, dynamic, and non-ionizing radiation, and it provides better visualization of microvascular perfusion. It can also be used in patients with renal insufficiency or allergy to gadolinium or iodine-based agents (5). Here, we report a case of CDC with emphasis on Sonazoid CEUS.

## 2 Case presentation

**Figure 1:**
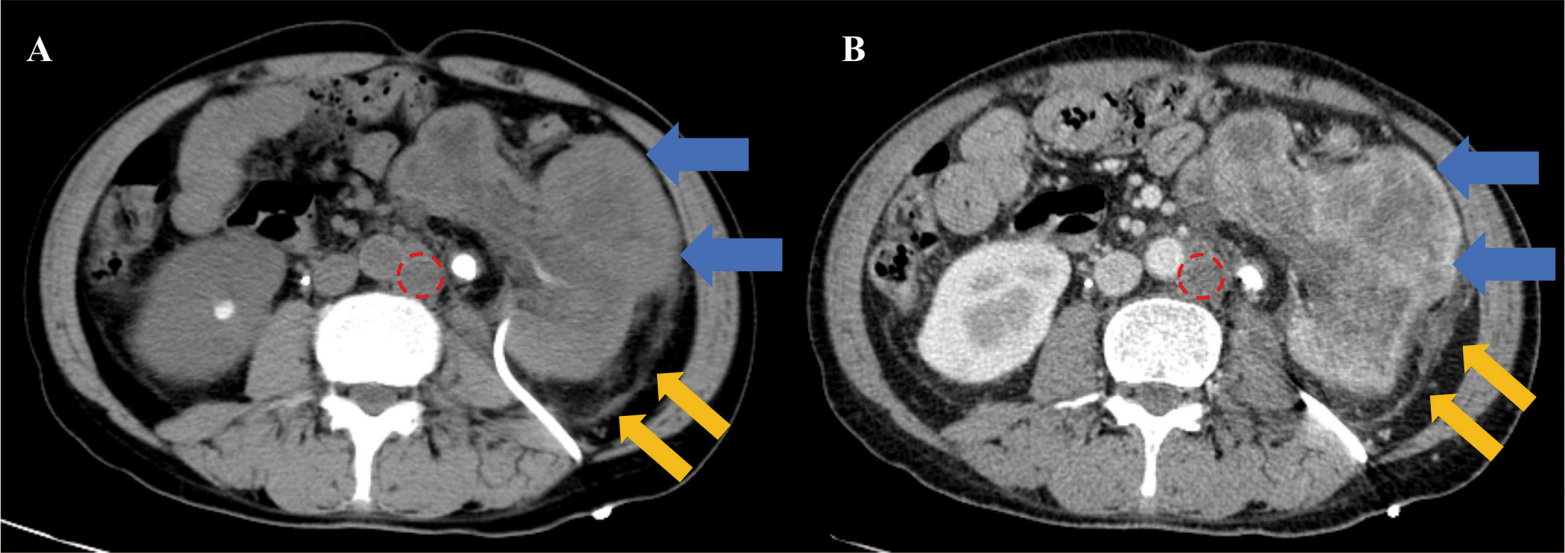
CT and CECT images. The left renal shows significant enlargement with distorted morphology, irregular dilation of renal pelvis and calyces, thinning of renal parenchyma, unclear demarcation of cortex and medulla, and heterogeneous enhancement (blue arrow). The perirenal fascia and the paracolic sulcus peritoneum on the left side are swollen (yellow arrow). The enlarged lymph nodes are observed beside the abdominal aorta (red circle). **(A)**, CT image. **(B)**, CECT image cortical period.

This study has been approved by the Ethics Committee of Sichuan Integrative Medicine Hospital (No. KY-M-2022-001). A 49-year-old male presents with a 6-month history of bilateral lumbar distension and pain, worsening on the left side with hematuria for 3 days. The patient began experiencing lumbar distension and pain 6 months ago, predominantly on the left side, without referred pain. No other specific symptoms were noted. He was admitted to another facility and diagnosed with bilateral renal calculi. A left nephrostomy was performed and a right ureteral stent was placed. Three days ago, the patient’s left lumbar distending pain intensified, accompanied by urinary frequency, incomplete urination, and gross hematuria. No family history. Physical examination revealed a nephrostomy tube on the left side of the waist without redness or swelling around the stoma. The drained urine was pale red and cloudy. No other abnormalities were found. Laboratory findings: total protein (TP): 47.7 g/dL (65-85 g/dL), C-reactive protein: 160 mg/dL (0-10 mg/dL), white blood cells: 14.31*10^9 g/dL (3.50-9.50 g/dL), CA12-5: 81.69 U/mL (<35 U/mL), urine red blood Cells: 850/μL (0-11/μL), urine bacteria: 3637 CFU/mL (0-23 CFU/mL). Laboratory tests indicated the presence of infection and mild elevation of tumor markers. The patient’s CT and contrast-enhanced CT (CECT) images were shown in **Figure 1**. Elbow venous injection of iohexol 80ml, injection rate 2.5 ml/s. Scan slice thickness 7.5 mm, reconstruction slice thickness 1.25 mm, 512 x 512 pixels. The CT and CECT diagnosis: left renal abscess with necrosis and exudation surrounded by reactive lymph node hyperplasia.

**Figure 2:**
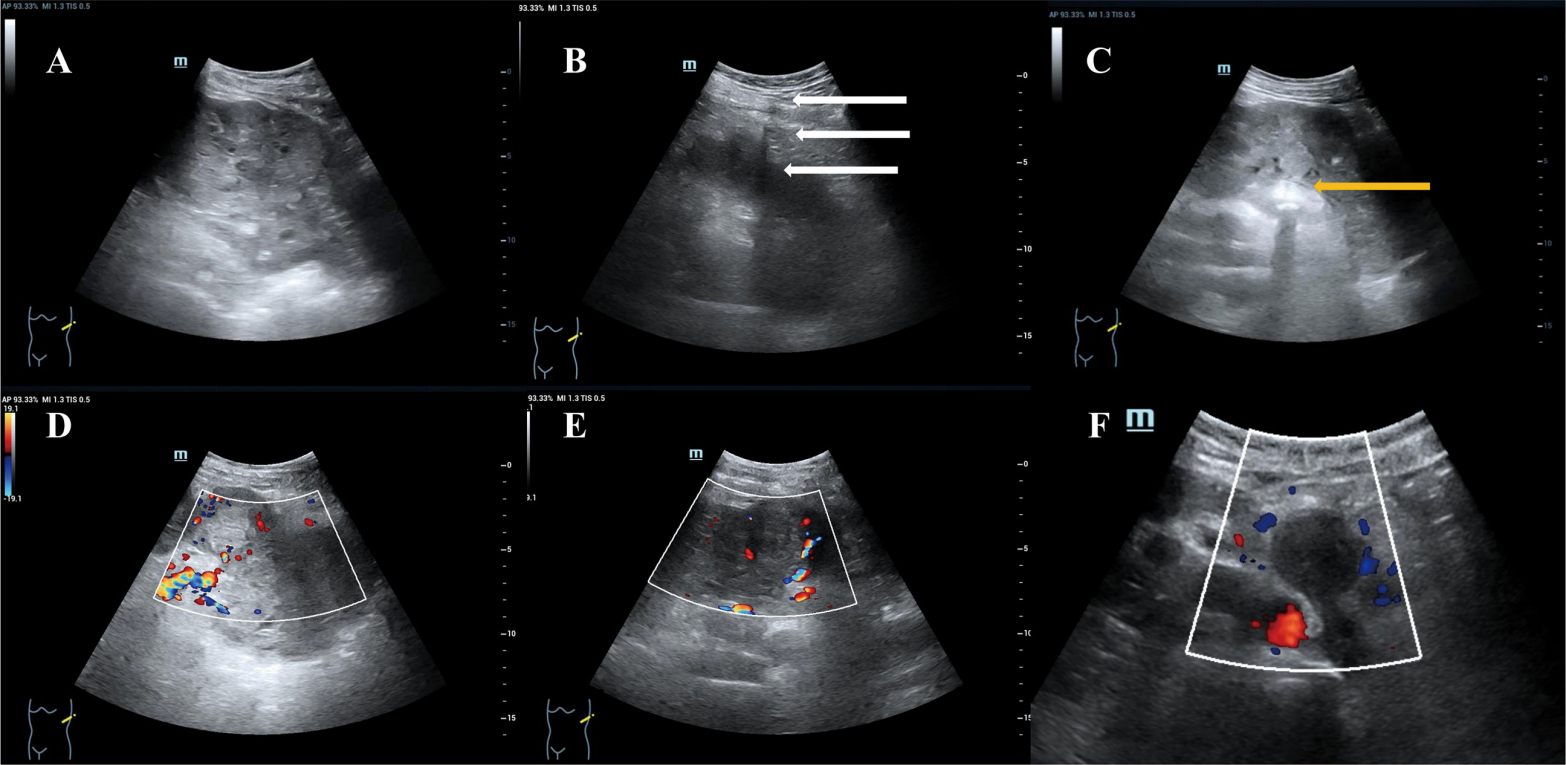
Two-dimensional ultrasound images. **(A)** The left renal showed abnormal morphology, with a tumor of mixed echogenicity observed internally, measuring 12x8 cm in size. The tumor has indistinct borders, an irregular shape, and displays heterogeneous internal echoes. **(B)** Drainage tube sound shadow (white arrow). **(C)** Left renal calculi (yellow arrow). **(D)** Renal hilar vascular structure was normal. **(E)** Blood signal in the tumor (Alder grade II). **(F)** Enlarged lymph nodes adjacent to the abdominal aorta.

**Figure 3:**
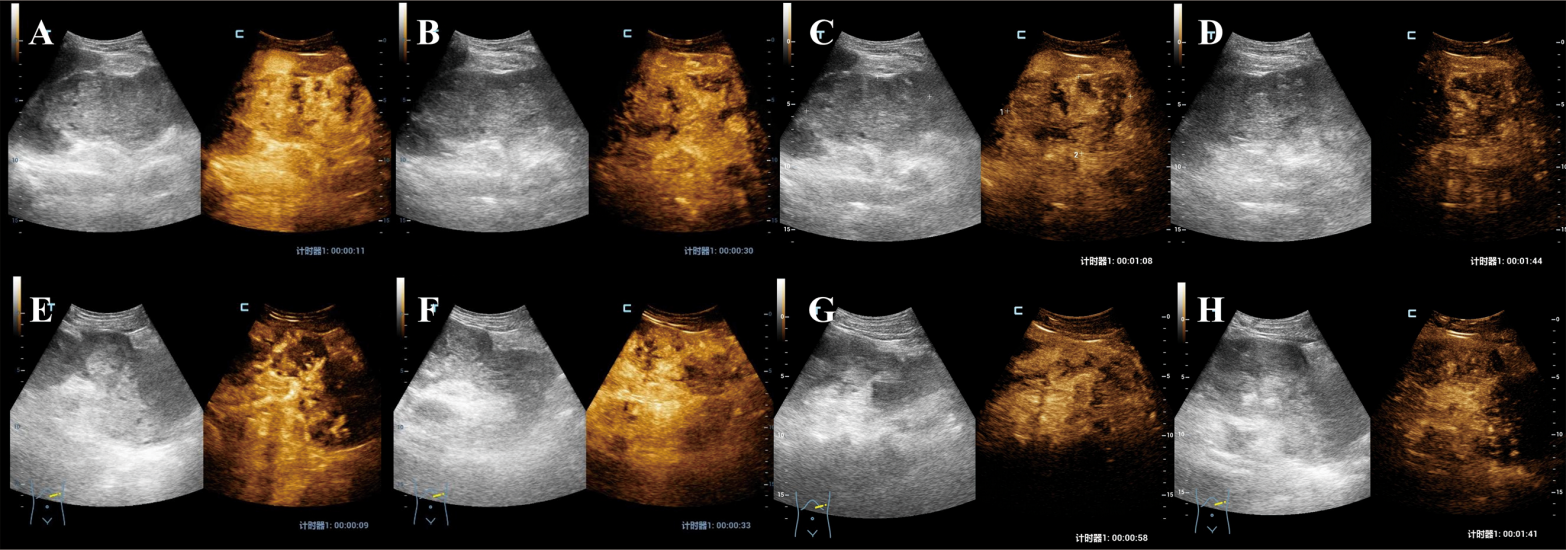
CEUS images. **(A–D)**, The upper part of the tumor. **(A, B)**, Cortical phase: At 11 seconds and 30 seconds, the tumor showed heterogeneous rapid and intense enhancement with fissure-like non-enhancing areas within. **(C)**, Medullary phase: 1 minute 8 seconds. The tumor shows persistent heterogeneous hyperenhancement with fissure-like non-enhancement within. **(D)**, Parenchymal washout phase: At 1 minute and 44 seconds, the tumor showed washout with hypoenhancement. **(D–G)**, The lower part of the tumor. **(E, F)**, Cortical phase: At 9 seconds and 33 seconds, the tumor showed heterogeneous rapid and intense enhancement with fissure-like non-enhancing areas within. **(G)**, Medullary phase: 58 seconds. The tumor shows persistent heterogeneous hyperenhancement with fissure-like non-enhancement within. **(H)**, Parenchymal washout phase: At 1 minute and 43 seconds, the tumor showed washout with hypoenhancement.

The patient’s two-dimensional ultrasound was shown in **Figure 2**. Due to the large size of the tumor, CEUS was performed in two separate sessions, targeting the upper and lower portions of the tumor. Each session involved a bolus injection of 0.4 ml of Sonazoid via the elbow vein, and the second injection was performed after the complete washout of the first session. The CEUS images were shown in **Figure 3**. In the early enhancement phase (cortical and medullary phases) of the left renal tumor, it showed overall heterogeneous hyperenhancement compared to the surrounding tissue, suggesting a hypervascular tumor. In the late enhancement phase (parenchymal washout phase), the tumor washout to quickly show hypoenhancement. Fissure-like non-enhancing areas were observed within the left renal tumor. The ultrasound and CEUS diagnosis: left renal neoplastic tumor with surrounding lymph node metastasis. (The primary tumor in the left renal originates from the collecting system.)

**Figure 4:**
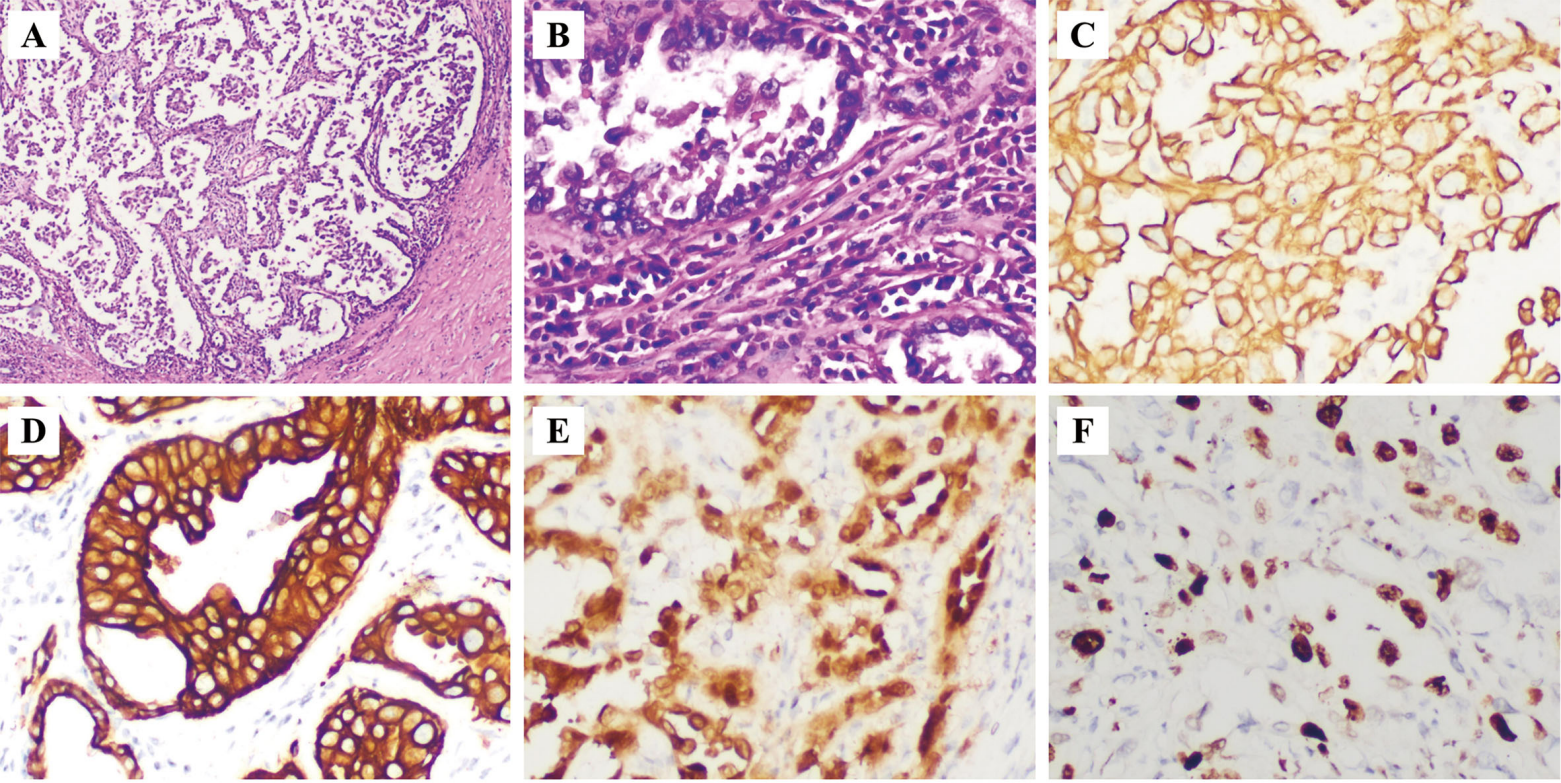
Pathology. **(A)** HE (x4); **(B)** HE (x20); **(C)** PCK (+, x20); **(D)** CK7 (+, x20); **(E)** Pax-8 (+, x20); **(F)** Ki-67 (about 60%, x20). HE: hematoxylin-eosin.

Intraoperatively, the left renal was found to be significantly enlarged with effusion, with abnormal morphology and a cauliflower-like appearance. The perirenal fascia was fibrotic, with indistinct anatomical planes and a firm texture. The perirenal fat capsule was markedly thickened to about 2 cm. A hard calculi was palpated at the junction of the left ureter and renal pelvis. Multiple enlarged, hard lymph nodes ranging from 2.5 to 3 cm in size were found fused together near the lower pole of the left renal and adjacent to the abdominal aorta. Postoperatively, upon gross examination of the left renal, the renal parenchyma was significantly thinned with a fish-flesh cut surface. The pathology are shown in **Figure 4**. Immunohistochemistry revealed: PCK(+)、EMA(+)、CK7(+)、34βE12(+)、Pax-8(+)、Vimentin(focal+)、p63 (-)、RCC(-)、CA1X(focal+)、p504S(focal+)、CD10(+)、CD117(-)、GATA-3(+). Ki-67 index was about 60% positive. The tumor presented as a tubular architecture, accompanied by irregularly infiltrating glands and a stroma with a desmoplastic reaction. The tumor cells displayed high-grade nuclear features. Pathological diagnosis: CDC of the renal, the abdominal lymph nodes were identified as metastatic carcinoma.

Four months postoperatively, follow-up revealed multiple liver metastases, bilateral adrenal metastases, multiple abdominal lymph node metastases, peritoneal metastasis, and pleural and peritoneal effusions, with the remaining right kidney free of tumor.

## 3 Discussion

Collecting duct carcinoma (CDC) of the renal is a rare epithelial tumor that accounts for only 0.4% to 2.0% of all renal carcinomas (2). The incidence rate has a male to female ratio of about 2:1 and can occur in various age groups, with an average age of onset at 55 years (3). At presentation, 30% to 50% of patients already have distant metastases (4). The etiology remains elusive, but potential triggers include genetic predisposition, smoking, obesity, hypertension, and immunocompromised status, which may be associated with alterations in the NF2 and CDKN2A genes (7). CDC originates from the epithelial cells of the collecting ducts in the renal medulla (8), located in the central part of the renal, and tends to grow invasively, with the potential for cystic degeneration, hemorrhage, and necrosis (9). CDC is characterized by its high malignancy, short disease course, and rapid progression, with a complex and variable clinical presentation. Common symptoms include hematuria, lumbar pain, and renal tumor (10). Laboratory tests lack specificity, with some patients exhibiting hematuria, erythrocytosis, decreased hemoglobin levels, elevated erythrocyte sedimentation rates, hyperglycemia, and hypercalcemia (10). Surgical resection is the primary treatment for localized tumors, while diffuse tumors are typically managed with a combination of chemotherapy and immunotherapy.

This patient underwent a left nephrostomy six months ago, and no tumor was found in the left renal at that time. However, six months later, the morphology of the left renal was completely abnormal. Laboratory tests upon admission indicated the presence of infection. Based on the patient’s medical history and the rapid progression of the disease, CT scan initially suggested the possibility of a renal abscess, and the lymph nodes were considered as inflammatory hyperplasia, leading to a misdiagnosis of this case. The two-dimensional ultrasound examination revealed the absence of normal renal imaging in the left renal region. Instead, a large mixed echoic tumor was observed, with indistinct borders, an irregular shape, and heterogeneous internal echoes. Through the visualization of drainage tubes and stones, as well as the detected renal hilum blood flow signals, it was determined that the tumor originated from the left renal. The overall appearance of the tumor resembled an enlarged renal, with no visible normal renal parenchyma and no tumor protruding beyond the renal contour. On further detailed examination using CEUS, the tumor in this case showed an expansive growth pattern with no clear renal borders. Renal abscesses typically show a ring-like or heterogeneous hyperenhancement in the early enhancement phase, with no enhancement in the abscess cavity (11). However, given that this patient’s disease course had lasted for nearly six months without the formation of an abscess cavity, which was rare, the possibility of an abscess was excluded. Furthermore, the tumor showed hyperenhancement in the early phase, which rapidly washed out in the late phase, consistent with the “fast-in and fast-out” pattern seen in common malignancies. Characteristic fissure-like non-enhancing areas were observed within the tumor, with regular shapes and arrangements, which were considered dilated renal collecting ducts rather than necrotic abscess cavities. CEUS based on harmonic ultrasound imaging technology significantly improves image contrast and resolution by leveraging the nonlinear effects of ultrasound waves, particularly in visualizing subtle structures and microvascular networks, improving the visualization of blood perfusion and intricate details within the tumor, thereby allowing for a more accurate assessment of its characteristics, including the fissure-like non-enhanced areas that resemble the calyceal structure and are of considerable diagnostic value. Therefore, combining the findings from two-dimensional ultrasound and CEUS, a highly malignant tumor originating from the left renal medulla was considered the most likely diagnosis, negating the CT diagnosis. Surgical resection ultimately confirmed the diagnosis of CDC. In the present case, we did not observe composite subtypes within the tumor. However, notable intratumoral heterogeneity was identified. This heterogeneity was reflected in the variable expression of markers such as PCK, EMA, CK7, 34βE12, and Pax-8 across different regions of the tumor, indicating diverse cellular phenotypes and potential differences in tumor cell biology. The observation of heterogeneity underscores the complex nature of renal cancers and suggests that intratumoral genetic and phenotypic diversity may contribute to the aggressive behavior and treatment resistance observed in these neoplasms. Further studies exploring the molecular underpinnings of this heterogeneity could provide insights into the pathogenesis and progression of renal cell carcinoma, potentially leading to more targeted and effective therapeutic strategies. In addition to differentiating from abscesses, this case required differential diagnosis from other conditions. Firstly, granulomatous nephritis has to be considered because the patient’s history of catheterization could lead to foreign body stimulation and granuloma formation. However, granulomatous nephritis typically shows hypoenhancement on CEUS, which is inconsistent with the appearance of CDC. Secondly, clear cell renal cell carcinoma (ccRCC) needed to be differentiated from CDC, ccRCC is the most common primary renal tumor. ccRCC has variable two-dimensional ultrasound echo patterns, often presenting as solid hypoechoic tumors, and it is frequently located outside the renal contour, with surrounding tissues compressed and displaced. CEUS of ccRCC typically shows heterogeneous hyperenhancement in the early phase, though a minority may demonstrate homogeneous hyperenhancement. ccRCC commonly has a pseudocapsule, and it may have hemorrhage or necrosis. During the washout phase, the enhancement can clear either rapidly or slowly (12). Furthermore, in early-stage tumors originating from the urothelium, they are typically observed to grow within the renal pelvis and show an infiltrative growth pattern. On CEUS, these tumors often show a rapid wash-in hyperenhancement. However, for larger, late-stage tumors where the surrounding tissue structures are difficult to distinguish, differentiating them from CDC can present challenges. In such cases, additional diagnostic modalities such as CT or MRI scan, as well as a comprehensive clinical assessment, may be necessary to establish an accurate imaging diagnosis.

The ultrasonographic appearance of CDC varies depending on its location, size, and morphology, predominantly presenting as a solid tumor with indistinct borders and an irregular shape. Study had reported (13)that on CT/MRI images, CDC typically shows an inhomogeneous low-density/abnormal signal tumor within the renal parenchyma, with unclear borders and an irregular shape. The tumor may contain calcifications, hemorrhage, necrosis, or cystic changes, resulting in density/signal inhomogeneity. The surrounding tissues are often compressed. On PET-CT images, CDC typically appears as an area of abnormally increased glucose metabolism in contrast to the surrounding normal tissue. In this case, no tumor was observed to extend beyond the kidney contour, and the overall growth pattern was expansive. CEUS showed a hypervascular tumor with rapid contrast agent uptake in the early phase, resulting in hyperenhancement. In the late phase, the enhancement rapidly washout, resulting in hypoenhancement. The overall enhancement pattern was heterogeneous, resembling the findings reported by Tang et al. (14) for CDC. Moreover, in combination with two-dimensional ultrasound diagnosis, it is more likely to be due to malignant tumors of the renal medulla than to other tumors. CEUS is of great importance in the diagnosis and treatment of this case.

In recent years, CEUS has been increasingly used in the diagnosis of renal tumors and has a high diagnostic value in solid renal tumors (15). Different types of solid renal tumors have specific characteristics on CEUS, which can improve the accuracy of diagnosis and differential diagnosis. In addition, for tumors of unknown etiology, CEUS can provide rich diagnostic information by examining the morphology, intensity, and perfusion pattern of the tumor enhancement.

## 4 Conclusion

CEUS can be helpful in the diagnosis of CDC. If a tumor has an enhancement pattern that is not typical of renal parenchymal origin, especially if fissure-like non-enhancing areas resembling renal calyces are observed, the possibility of renal medullary origin CDC should be considered. The real-time and dynamic observation provided by CEUS may increase confidence in the diagnosis of CDC.

## Data Availability

The raw data supporting the conclusions of this article will be made available by the authors, without undue reservation.
